# Exosc9 Initiates SUMO-Dependent lncRNA TERRA Degradation to Impact Telomeric Integrity in Endocrine Therapy Insensitive Hormone Receptor-Positive Breast Cancer

**DOI:** 10.3390/cells12202495

**Published:** 2023-10-20

**Authors:** Maram Quttina, Kacie D. Waiters, Ashfia Fatima Khan, Samaneh Karami, Anthony S. Peidl, Mariam Funmi Babajide, Justus Pennington, Fatima A. Merchant, Tasneem Bawa-Khalfe

**Affiliations:** 1 Center for Nuclear Receptors & Cell Signaling, Department of Biology & Biochemistry, University of Houston, 3517 Cullen Blvd, SERC Bldg, Rm 3010, Houston, TX 77204-5056, USAakhan70@central.uh.edu (A.F.K.);; 2 Engineering Technology College of Technology, University of Houston at Sugarland, 13850 University Blvd, SAB1 Bldg, Rm 348, Sugarland, TX 77479, USA

**Keywords:** Exosc9, HP1-alpha, TERRA, SUMO, telomeric R-loops

## Abstract

Long, noncoding RNAs (lncRNAs) are indispensable for normal cell physiology and, consequently, are tightly regulated in human cells. Yet, unlike mRNA, substantially less is known about the mechanisms for lncRNA degradation. It is important to delineate the regulatory control of lncRNA degradation, particularly for lncRNA telomeric repeat-containing RNA (TERRA), as the TERRA-telomere R-loops dictate cell cycle progression and genomic stability. We now report that the exosome complex component Exosc9 degrades lncRNA TERRA in human mammary epithelial cells. Heterochromatin protein 1 alpha (HP1α) recruits Exosc9 to the telomeres; specifically, the SUMO-modified form of HP1α supports interaction with Exosc9 and, as previously reported, lncRNA TERRA. The telomeric enrichment of Exosc9 is cell cycle-dependent and consistent with the loss of telomeric TERRA in the S/G2 phase. Elevated Exosc9 is frequently observed and drives the growth of endocrine therapy-resistant (ET-R) HR+ breast cancer (BCa) cells. Specifically, the knockdown of Exosc9 inversely impacts telomeric R-loops and the integrity of the chromosome ends of ET-R cells. Consistently, Exosc9 levels dictate DNA damage and the sensitivity of ET-R BCa cells to PARP inhibitors. In this regard, Exosc9 may serve as a promising biomarker for predicting the response to PARP inhibitors as a targeted monotherapy for ET-R HR+ BCa.

## 1. Introduction

### Telomere Function and Dysfunction

Chromatin is highly enriched with regulatory RNAs, including long, noncoding RNAs (lncRNA). A growing number of studies highlight the critical roles of chromatin-bound lncRNA in normal cellular physiology and, adversely, in pathophysiology. Telomeric repeat-containing RNA (TERRA) is an example of a unique lncRNA that is required to ensure the integrity of chromosome ends or telomeres. Telomeres are compact heterochromatic regions that include repetitive TTAGGG double-stranded DNA of 5000–20,000 bases in length and a smaller 100–300 G-rich single-stranded DNA. In order to promote the replication and maintenance of this protective structure, the telomeres interact with a select repertoire of molecules like lncRNA TERRA and nucleoprotein enzyme telomerase. The loss of telomeres subjects the chromosome tip to attack from DNA damage repair (DDR) machinery and initiates cell cycle blockage. Seminal studies establish that the induction of telomerase allows cells, particularly cancer cells, to bypass this growth crisis, but the role of TERRA is not as well defined.

TERRA is transcribed from the promoters on the subtelomeric region of the C-rich DNA strand. Consistently, TERRA molecules are heterogeneous with unique chromosome-specific subtelomeric sequences and variable telomeric UUAGGG repeats. TERRA binds telomeric DNA in trans to form RNA-DNA hybrid chromatin structures called telomeric R-loops [1]. Hence, telomeric R-loops are unique in contrast to ubiquitous cis R-loops that commonly form between native RNA and its template DNA. Interestingly, TERRA lncRNA levels are dynamic and tightly regulated during cell cycle progression. TERRA is enriched in HeLa cells during the G1- and early S phase and is dramatically reduced in the transition from the S to the G2 phase [2]. Yet, it is unclear what directs cell-cycle-dependent TERRA clearance, particularly in human cells.

TERRA lncRNA interacts with multiple proteins at the telomeres; this includes but is not limited to telomerase, telomere-associated shelterin protein complex, chromatin remodelers, and DNA damage repair components [3,4,5,6]. We and others have shown that heterochromatin protein 1 alpha (HP1α) interacts with TERRA to maintain the heterochromatin formation at the telomeres [7,8]. HP1α undergoes post-translational modifications (PTMs), including SUMOylation, and the deSUMOylase enzyme SENP7 regulates the SUMOylation state of HP1α. Recently, we reported that the SUMOylation of HP1α enhances its interaction with TERRA. Currently, it is unknown if and how the SUMO modification state of HP1α directs TERRA and telomere biology. Surprisingly, TERRA also interacts with the RNA exosome complex, including exosome component 9 (Exosc9) in humans [9]. RNA exosome complex is the major mRNA degradation machinery and is highly conserved among different species. Recent studies suggest multiple subunits of the RNA exosome contribute to the degradation of lncRNA in R-loops [10]. However, the ability of Exosc9 to direct TERRA degradation is currently unknown.

Aberrant TERRA levels contribute to disease onset. For example, TERRA accumulates within immunodeficiency, centromeric instability, and facial anomalies (ICF) syndrome to perturb telomere replication, accelerate telomere loss, and initiate cellular senescence [11,12]. The dysregulation of TERRA is also frequent in multiple types of cancer but with different results.

In several telomerase-positive cancers, TERRA is a tumor suppressor [13,14,15] and is consistent with TERRA’s function to inhibit telomerase activity [6]. In murine mammary epithelial cells, TERRA represses the expression of epithelial-mesenchymal transition genes [16], highlighting the protective role of TERRA against metastatic transformation. Yet, TERRA’s definitive role in breast cancer (BCa) is unclear, particularly the predominant hormone receptor-positive (HR+) BCa subtype. Approximately 70% of patients diagnosed with HR+ BCa are treated with endocrine therapy (ET) as the first-line strategy for treatment. However, 30–40% of HR+ BCa patients exhibit intrinsic or acquired ET resistance (ET-R) and readily progress to incurable metastatic disease. As targeted therapy options continue to expand, it is critical to establish biomarkers to stratify potential drug responders from non-responders.

This study delineates a mechanism for regulating lncRNA degradation on chromatin. SUMO-HP1α binds to the RNA degradation component Exosc9 and recruits it to the chromatin regions during the cell cycle, specifically at the S/G2 phase. At the telomeres, Exosc9 is enriched during S/G2, and this enrichment is enhanced by the presence of SUMO-HP1α and is critical for regulating TERRA degradation during the cell cycle. This study also shows that the disruption of the cell cycle regulation of TERRA accumulates DNA damage at the telomeres in ET-R BCa. Consistently, Exosc9 levels predict the sensitivity of ET-R BCa cells to the targeted PARP inhibitor, olaparib.

## 2. Materials and Methods

### 2.1. Cell Cultures, Transfections, and siRNA Treatments

MCF10-2A cells were grown in DMEM: F12 media supplemented with insulin, EGF, cholera toxin, hydrocortisone, 5% horse serum, and 1% penicillin/streptomycin. Tamoxifen-sensitive MCF7 (TamS-7) cells, the triple-negative breast cancer cell line (MDA-MB-231), and endocrine-resistant cells (Tamoxifen-resistant MCF7 (TamR-7), GI-101A, and GILM2) were cultured in DMEM high-glucose media with 10% FBS and 1% penicillin/streptomycin. TamR-7 cells were generated, as previously described [17].

Overexpression studies were carried out on the MCF10-2A cells using an empty pcDNA vector, pcDNA3-nV5-HP1α (wt-HP1α), pcDNA3-nHA- SUMO3-fused HP1α (SUMO-HP1α), pcDNA3-nV5-HP1α (K to R HP1α mutant), and pCMV3-N-OFPSpark-EXOSC9 (Sino Biological). Transient transfection was performed with Lipofectamine 2000, according to the manufacturer’s instructions (Invitrogen).

siRNA knockdown studies were performed in MCF10-2A cells or TamR-7 cells using commercially available nontargeting siRNA (siNT), EXOSC9 siRNA (pool purchased from Dharmacon), or SENP7 siRNA (pool purchased from Dharmacon). Dharmafect-2 reagent was used for siRNA transfection, as directed (GE Life Sciences, Chicago, IL, USA).

### 2.2. Chromatin-Bound Protein Immunoprecipitation

Cells were collected, harvested with cold skeleton buffer (CSK), and lysed using immunoprecipitation (IP) lysis buffer supplemented with protease inhibitor (Roche, Basel, Switzerland) and N-ethylmaleimide (Sigma-Aldrich, St. Louis, MO, USA). Chromatin fractions were used for protein immunoprecipitation, as described before [18]. Protein-A agarose beads (Millipore, Burlington, MA, USA) were added and incubated with the IP samples. Input and immunoprecipitated samples were eluted with Laemmli sampling buffer, boiled, and subjected to Western blot analysis.

### 2.3. SDS-PAGE & Western Blot Analysis

Input and CB/IP samples were separated on SDS-page gels and blotted to PVDF membranes (BioRad, Hercules, CA, USA). Membranes were blocked and probed overnight with HP1α antibody (Novus Biologicals, Centennial, CO, USA; NB110-40623), EXOSC9 antibody (Santa Cruz Biotechnology, Santa Cruz, CA, USA; sc-271815), SUMO2/3 antibody (Abcam, Cambridge, UK; ab3742), SENP7L antibody [17], or Cyclin B1 antibody (Santa Cruz Biotechnology; sc-245). Proteins were detected on membranes using the chemiluminescence Western Lighting Plus-ECL reagent (PerkinElmer, Waltham, MA, USA).

### 2.4. Chromatin Immunoprecipitation

ChIP-IT^®^ Express (Active Motif, Carlsbad, CA, USA) kit was used for the preparation of ChIP samples. Briefly, MCF10-2A or TamR-7 cells were fixed with 1% formaldehyde (Sigma, St. Louis, MO, USA) and quenched with 1x glycine. After several washes, the cells were pelleted and lysed. Chromatin was sheared, and DNA was sonicated using (Diagenode, Bioruptor, Sparta, NJ, USA) to generate 200- to 500-bp fragments. EXOSC9 antibody (Abcam), HP1α antibody (Abcam), EXOSC10 antibody (Santa Cruz Biotechnology), EXOSC4 antibody (Santa Cruz Biotechnology), or γH2AX antibody (Millipore) were used for chromatin immunoprecipitation. Protein G magnetic beads were added to ChIP samples and incubated overnight. Chromatin was eluted and treated with Proteinase K.

TELO-ChIP experiments utilized chromatin immunoprecipitation, followed by evaluating telomeric enrichment utilizing TELO primers on the eluted DNA (Supplemental Table S1).

### 2.5. DNA-RNA Chromatin Immunoprecipitation (DRIP)

ChIP-IT^®^ Express (Active Motif) was used as described. Following the generation of bp fragments, DNA-RNA Hybrid mAb (Clone S9.6) antibody (Active Motif) was used for chromatin immunoprecipitation.

### 2.6. Real-Time PCR

Total RNA was prepared using PureLink RNA Mini Kit (ThermoFisher Scientific, Waltham, MA, USA) and converted to cDNA with an iScript cDNA Synthesis Kit (BioRad), according to the manufacturer’s instructions. Primer sequences are listed in (Supplemental Table S1; IDT). RT-PCR was performed using the SsoAdvanced universal SYBR^®^ Green supermix (Biorad) and 7500 Fast Real-Time PCR System (Applied Biosystems, Waltham, MA, USA). Data were normalized to the reference gene (β-actin) and analyzed by the ΔΔCT method. As per the MIQE guidelines, additional endogenous controls, GADH and 18S, were used.

### 2.7. Metaphase Spread Preparations

MCF10-2A cells were grown until full confluence. Cells were then incubated with 0.25 µg/mL Colcemid (GIBCO™, Kary MAX^®^, #15212-012) for 3–4 h. Cells were collected by trypsinization and resuspended in a prewarmed 0.075 M potassium chloride (KCl) solution at 37 °C for 20 min. After centrifugation, the cells were fixed in a cold (methanol: acetic acid) solution, which was prepared in a 3:1 ratio, respectively. Fixed cells were dropped onto a microscopic slide, held on steam for 30 s and left to air-dry. Slides were stored in 100% ice-cold ethanol for subsequent FISH preparations.

### 2.8. Telomeric Fluorescent In Situ Hybridization

Metaphase spread slides were washed in 1X PBS and were immersed in pepsin solution for 2 min at 37 °C. Slides were washed twice with 1X PBS and were subsequently dehydrated in 70%, 90%, and 100% ethanol, respectively, for 5 min each and left to dry. A total of 20 μM of 500 nM Cy3-conjugated telomeric PNA probe (PNA Bio, Newbury Park, CA, USA) in hybridization buffer (70% formamide, 0.25% blocking reagent Roche, and 10 mM Tris HCl pH 7.4) was added to the slides. Slides were covered and denatured at 85 °C for 10 min. Slides were then incubated for 2 h at RT in the dark in a humid chamber. Slides were immersed in wash solution (2X SSC, 0.1% Tween-20) to remove the cover and were washed with wash solution twice at 55~60 °C for 10 min and once at room temperature. Slides were mounted with Vectashield (Vector Labs, Newark, CA, USA), and images were captured using the Olympus BX51 microscope. Telomere morphology was evaluated by looking for aberrancies previously noted by others [17,19]. To eliminate bias, three individuals were independently evaluated for telomere morphology on 9–11 random microscope images for each cell line in two biological replicate studies.

### 2.9. Combined Immunofluorescence & Fluorescent In Situ Hybridization

Intact cells were fixed with 4% formaldehyde in PBS at 4 °C and permeabilized with PBS 0.1% Triton X-100 at room temperature. Slides were then blocked with 5% (*w*/*v*) BSA in 1X PBS for 30 min at room temperature and then incubated with Exosc9 (Abcam) or γH2AX antibody (Millipore) diluted in 1% BSA in PBS solution for 1 h at room temperature. Slides were blocked again and then incubated with the secondary antibody for 1 h at room temperature. After immunostaining, telomeric Fluorescence in situ hybridization (FISH) was performed using a telomeric PNA probe (PNA Bio), as described above. Slides were mounted with Vectashield (Vector Labs), and images were captured using the Olympus BX51 microscope. Positive colocalization was identified as yellow foci in the merged IF images. A total of 29 random microscope images from two biological replicates were evaluated.

### 2.10. Double Thymidine Block and Cell Cycle Analysis

Double thymidine block was performed on MCF10-2A cells. Cells were seeded at 30% confluence. Cells were grown for 24 h and then treated with 2 mM thymidine for 18 h. After the first block, cells were released for 9 h by the addition of fresh media. Cells were blocked again with 2 mM thymidine for 17 h. Cells were then released and collected at 0 h, 4 h, and 6 h, respectively. Propidium iodide (PI) staining and the cellometer (Nexcelom, Lawrence, MA, USA) were used to assess cell cycle synchronization according to the manufacturer’s instructions.

### 2.11. In Vitro Exoribonuclease Assay

Total RNA was extracted using PureLink RNA Mini Kit (ThermoFisher Scientific), and RNA concentration was measured using Nanodrop. Exoribonuclease assay was prepared using 1 µg of RNA in the reaction buffer (10 mM Tris HCL [PH 8.0], 10 mM DTT, 50 mM KCL, 5 mM MgCl_2_, 1 U/µL RNase inhibitor) and incubated with/without EXOSC9 recombinant protein (Novus Biological) at 37 °C for different time periods (0 min, 15 min, 30 min, 45 min, and 60 min). The reactions were terminated with RNA purification buffer. RT-qPCR was carried out using the standard curve method, whereby the known concentrations of extracted RNA were prepared, converted to cDNA, and amplified with fibronectin- and TERRA-specific primers (Supplemental Table S1; IDT).

### 2.12. In Vitro SUMOylation

The in vitro SUMOylation of HP1α recombinant protein was performed, as previously described [20]. Following SUMOylation, HP1α was immunoprecipitated from unmodified HP1α and SUMOylated HP1α. Immunoprecipitated protein was immobilized on agarose beads and incubated with Exosc9. Eluted protein was analyzed using SDS-PAGE gel. The interaction of Exosc9 with unmodified HP1α was quantified using densitometry with ImageJ version 1.53t.

### 2.13. Tumor Spheroid Studies

Tumor spheroid formation was performed, as previously described [21]. The 24 h siRNA (nontargeting and Exosc9-targeted siRNA) treated TamR-7 cells were dissociated to generate a single-cell suspension. A total of 20,000 single cells were seeded in non-adherent conditions in an ultra-low attachment 6-well plate. After 24 h, the tumorspheres were treated with increasing doses of olaparib (2 μM, 5 μM, 10 μM, 20 μM, and 50 μM, respectively). Spheroid count and diameter were evaluated following drug treatment. Tumorsphere images were acquired using the Olympus BX51 microscope. To eliminate bias, three individuals independently evaluated the spheroid images. The total number of spheroids on random images was counted from 3–4 biological replicates. By using the scale bar in the accompanying software, the spheroid diameters were recorded from random images, and statistical analysis was performed.

### 2.14. CB/IP Densitometry Analysis

Densitometry analysis was performed using ImageJ version 1.53t. Specifically, CB/IP densitometry represents a fraction of the intensity of the bands for a protein of interest normalized to the immunoprecipitated protein.

### 2.15. Statistical Analysis

GraphPad Prism (GraphPad Software version 9) was used to perform the statistical analysis. A Student’s *t*-test or one-way ANOVA with Tukey’s post-hoc tests was used to test for significance (*p* < 0.05).

## 3. Results

### 3.1. Interaction with SUMOylated HP1α Supports the Telomeric Enrichment of Exosc9

**Figure 1:**
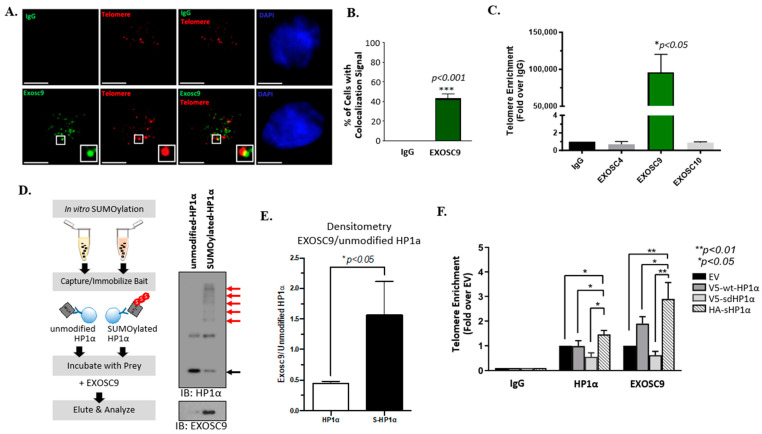
Exosc9 is enriched at telomeres by interacting with SUMOylated HP1α. (**A**) Combined IF-FISH was conducted using Exosc9 antibody (green) and telomere probe (red) in MCF10-2A cells. The arrowheads indicate the colocalization of Exosc9 with telomeres. Scale bars: 10 µm (main images) and 1 µm (inset). IgG was used as a negative control to exclude the non-specific fluorescent signals. (**B**) Data from (**A**) were quantified and plotted. Data expressed as percentage ± SEM (Student’s *t*-test; *** *p* value < 0.001). (**C**) ChIP-qPCR was performed to confirm IF-FISH data for Exosc9 and to check for the presence of Exosc4 and Exosc10 at telomeres. IgG served as a negative control. ChIP values were normalized to mean % input and fold enrichment was compared to the IgG. Error bars correspond to mean ± SEM from three independent experiments. Student’s *t*-test was used to test for statistical significance. (**D**,**E**) Immobilized cell-free bait (unmodified and SUMOylated HP1α) captures prey (Exosc9). Western blot shows Exosc9 interaction with immunoprecipitated SUMO-modified (red arrows) and unmodified (black arrow) HP1α recombinant protein. (**F**) MCF10-2A cells were transfected with EV, wild-type HP1α (V5-HP1α wt), SUMO-deficient mutant HP1α (V5-SdHP1α), or SUMO3-HP1α (HA-SHP1α). ChIP of HP1α or EXOSC9. ChIP was quantified by qPCR to check whether HP1α SUMOylation is required; ChIP-qPCR was performed to assess the enrichment of HP1α and Exosc9 at telomeres. ChIP values were normalized to mean input, and fold enrichment was compared to the EV. The error bars correspond to mean ± SEM from three independent experiments. ANOVA and Tukey’s post-hoc tests were used to test for statistical significance. Transfection was validated by Western blot.

Exosc9 is a core component of the exosome RNA degradation complex [22]. In order to understand the function of Exosc9 in relation to telomeres, we first evaluated Exosc9 enrichment at the telomeres. A total of 40% of noncancerous mammary epithelial cells exhibit colocalization between Exosc9 and telomeres (Figure 1A,B). IgG was used as a negative control to eliminate any non-specific fluorescent signals.

We next conducted TELO-ChIP experiments to evaluate the enrichment of the critical components of the RNA degradation system, specifically at the telomeric region. With IgG and Alu as the negative controls (Supplemental Figure S1A), we observed the significant enrichment of Exosc9 at the telomeres (Figure 1C). In contrast, two additional exosomal proteins, Exosc4 or Exosc10, were not recruited to the telomeric region of mammary epithelial cells (Figure 1C). This result suggests that Exosc9 is solely present at the telomeres, independently of the whole exosome complex. We further explored whether Exosc9 enrichment at telomeres requires interaction with specific chromatin-binding proteins. Others report a direct interaction between HP1α and exosomal components, including Exosc10 [23]. Interestingly, this interaction is highly enhanced when HP1α is SUMOylated. We performed an in vitro SUMOylation assay of HP1α followed by a bait-prey interaction study using the recombinant Exosc9 protein (Figure 1D). Surprisingly, we observed that the SUMOylated form of HP1α interacts more readily with Exosc9 (Figure 1D,E).

In order to show whether HP1α SUMOylation is required for Exosc9 recruitment at telomeres, we evaluated telomeric enrichment of HP1α and Exosc9 in MCF10-2A cells. Constructs that were previously characterized [8,18,24] to express WT-HP1α (V5-wt-HP1α), SUMO-deficient HP1α (V5-sd HP1α), and SUMOylated HP1α (HA-s HP1α) were used (Supplemental Figure S1B). Interestingly, the overexpression of SUMOylated HP1α enriches Exosc9 and HP1α at telomeres when compared to WT-HP1α or SUMO-deficient HP1α (Figure 1F). These results indicate that the RNA degradation exosome component Exosc9 is recruited to telomeres via interaction with the chromatin remodeler protein HP1α in a SUMO-dependent manner.

### 3.2. Telomeric Enrichment of Exosc9, like TERRA, Is Cell Cycle-Dependent

**Figure 2:**
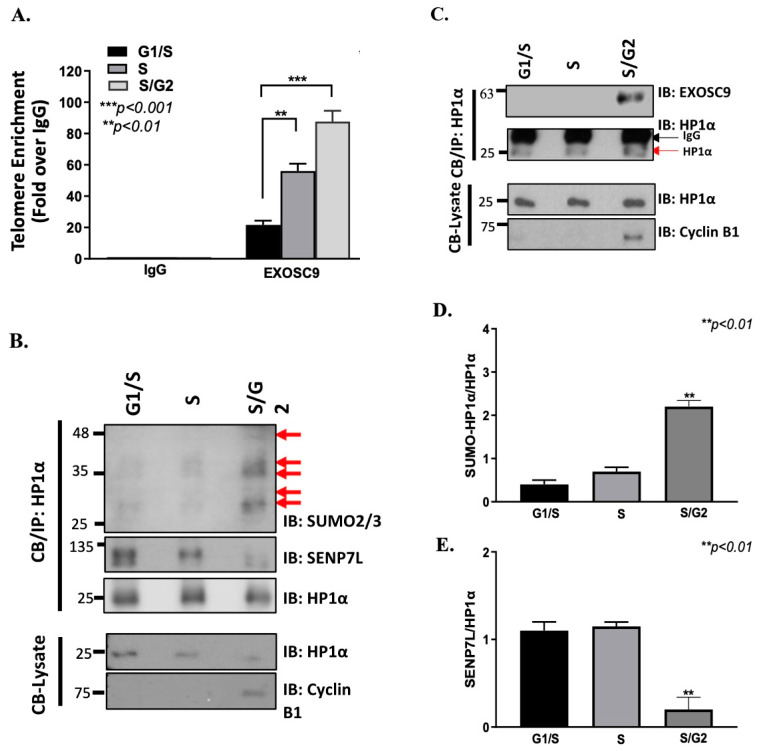
Exosc9 is enriched at telomeres in a cell cycle-dependent manner. (**A**) MCF10-2A cells were synchronized using a double thymidine block. Cells were collected in the G1/S, S, and S/G2 phases. ChIP-qPCR was performed to assess the enrichment of Exosc9 at telomeres during the cell cycle. IgG served as a negative control. ChIP values were normalized to mean % input, and fold enrichment was compared between different cell cycle stages. Error bars correspond to mean ± SEM from three independent experiments. ANOVA and Tukey’s post-hoc tests were used to test for statistical significance. (**B**) MCF10-2A cells were synchronized using a double thymidine block. CB/IP was performed using HP1α for the IP. The input and IP samples were then immunoblotted for HP1α and Exosc9. Cyclin B1 was used as a marker for cell cycle synchronization. Red arrows distinguish between the IgG and HP1α bands. (**C**) MCF10-2A cells were synchronized using a double thymidine block. CB/IP was performed using HP1α for the IP. The input and IP samples were then immunoblotted for HP1α, SUMO2/3, and SENP7L. Cyclin B1 was used as a marker for cell cycle synchronization. The red arrows represent SUMO-conjugates of HP1α. (**D**,**E**) Blots were analyzed by densitometry using ImageJ, normalized to HP1α, and graphed as the mean ± SEM. ANOVA and Tukey’s post-hoc tests were used to test for statistical significance.

TERRA clearance is required during DNA replication to maintain telomeric integrity [25]. In order to test this, we synchronized MCF10-2A cells using a double thymidine block, and we collected the cells after release into the cell cycle at 0 h (G1/S), 4 h (S), and 8 h (S/G2). Cell-cycle synchronization was confirmed using PI staining and a cellometer (Supplemental Figure S2A,B). Consistently, we also found that TERRA levels are significantly decreased within S/G2 in the noncancerous mammary epithelial cells (Supplemental Figure S2C). However, the mechanism of TERRA degradation remains unknown. In Figure 1, we showed that Exosc9 is present at the telomeres. Hence, we sought to determine whether Exosc9 is enriched at telomeres in a cell cycle-dependent manner. Interestingly, we found that synchronized cells in S/G2 show more telomeric enrichment of Exosc9 compared to synchronized cells in the G1/S or S phase (Figure 2A). This indicates that the presence of Exosc9 at telomeres is transient and analogous to low TERRA levels in the S/G2 phase.

As previously published in our systems [24], HP1α is enriched at the telomeres (Figure 1F). In addition, SUMOylated HP1α is readily telomere-enriched and associates with TERRA [8] and Exosc9 (Figure 1D) more than the unmodified form. Hence, we furthered our study by determining whether the HP1α-Exosc9 interaction occurs in a cell cycle-dependent manner. Endogenous chromatin-bound HP1α was isolated from synchronized MCF10-2A cells, and protein expression was observed to determine the HP1α-Exosc9 interaction at different cell cycle phases. Surprisingly, HP1α-Exosc9 interaction is uniquely demonstrated in S/G2 (Figure 2B). We also speculated that HP1α SUMOylation is required for the recruitment of Exosc9 to chromatin during the cell cycle. HP1α is readily SUMOylated at S/G2, and this is accompanied by the loss of the interaction between HP1α and SENP7L (Figure 2C–E). This finding supports our previous report in which it was found that the deSUMOylase enzyme SENP7L regulates the SUMO-modification of HP1α in mammary epithelia [26]. Cyclin B1 was used to validate cell cycle synchronization, which is only elevated in the S/G2 phase (Figure 2B,C). Collectively, our data highlight that SUMOylation combines chromatin remodeling and RNA surveillance to co-operatively regulate the ncRNA levels on chromatin.

### 3.3. Exosc9 Degrades TERRA lncRNA

**Figure 3:**
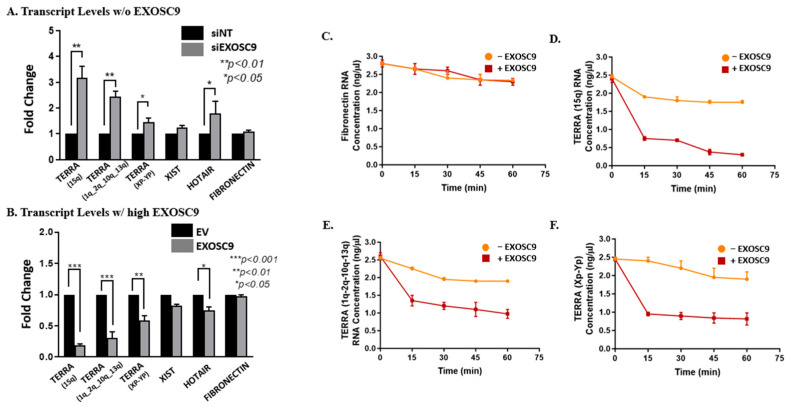
Exosc9 Drives TERRA Degradation. (**A**) MCF10-2A cells were transfected with nontargeting siRNA or Exosc9 siRNA.RT-PCR was carried out using primers corresponding to TERRA (1q-2q-10q-13q), TERRA (15q), TERRA (Xp-Yp), XIST, HOTAIR, and fibronectin mRNA. CT values were normalized to β-actin, and fold change was compared to nontargeting siRNA. Error bars correspond to mean ± SEM from three independent experiments. Student’s *t*-test was used to test for statistical significance. (**B**) MCF10-2A cells were transfected with EV or OFPSpark-Exosc9. RT-PCR was carried out using primers corresponding to TERRA (1q-2q-10q-13q), TERRA (15q), TERRA (Xp-Yp), XIST, HOTAIR, and fibronectin mRNA. CT values were normalized to β-actin, and fold change was compared to EV. Error bars correspond to mean ± SEM from three independent experiments. Student’s *t*-test was used to test for statistical significance. In vitro, an exosomal activity assay was performed using 1 µg of RNA in a specific reaction buffer in the presence/absence of Exosc9 recombinant protein and was incubated at 37 °C. Samples from (0 min. 15 min, 30 min. 45 min, and 60 min) were analyzed using absolute-qPCR with (**C**) fibronectin-specific primers. (**D**) TERRA (15q)-specific primers. (**E**) TERRA (1q-2q-10q-13q)-specific primers. (**F**) TERRA (Xp-Yp)-specific primers. Error bars correspond to mean ± SEM from two independent experiments.

It has been reported that core exosome subunits can independently degrade RNA molecules [27]. Hence, we postulated that Exosc9 regulates TERRA degradation. By utilizing knockdown/overexpression studies in MCF10-2A cells (Supplemental Figure S3), we evaluated TERRA expression by incorporating Exosc9-siRNA (siExosc9) for knockdown and OFP-SPARK Exosc9 plasmids for overexpression. The TERRA expression levels markedly increased in the absence of Exosc9 when compared to the nontargeting siRNA (siNT) (Figure 3A and Supplemental Figure S4A,B). Inversely, the overexpression of Exosc9 shows a significant decrease in TERRA expression when compared to EV (Figure 3B and Supplemental Figure S4C,D). This pattern is consistent with other lncRNAs, such as XIST and HOTAIR, where the expression levels of both lncRNAs increase in the absence of Exosc9 and decrease in the presence of Exosc9 (Figure 3A,B).

Additionally, we further assessed Exosc9’s ability to degrade lncRNA by transfecting noncancerous mammary epithelial cells (MCF10-2A) with V5-wt-HP1α and HA-sHP1α, our SUMOylated HP1α construct. Consistently, TERRA levels are significantly decreased in the presence of SUMOylated HP1α (Supplemental Figure S4E). These results indicate that SUMOylated HP1α has a critical role in recruiting Exosc9 to telomeres to initiate TERRA degradation. We then tested whether Exosc9 directly degrades TERRA. Using an in vitro degradation assay, endogenous RNA extracts from MCF10-2A cells were incubated with recombinant Exosc9 for various time intervals. Interestingly, TERRA transcript levels are incrementally reduced in the presence of recombinant Exosc9 protein by increasing the incubation time (Figure 3D–F). Contrarily, mRNA levels of fibronectin are not affected by the presence or absence of Exosc9 (Figure 3C). This suggests that Exosc9 exclusively degrades TERRA molecules. Collectively, our findings indicate that Exosc9 directly drives TERRA degradation and ultimately has a critical role in regulating lncRNA degradation.

### 3.4. Elevated Exosc9 with Endocrine Therapy Supports Aggressive BCa Growth

**Figure 4:**
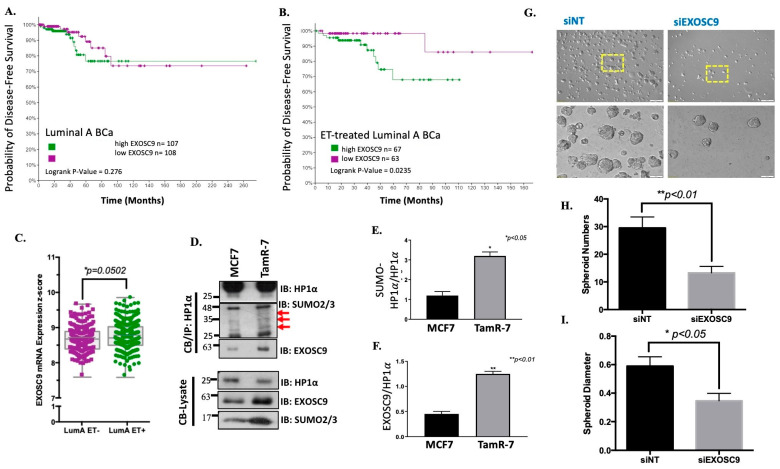
The Breast Invasive Carcinoma TCGA PanCancer Atlas dataset was utilized to identify luminal A breast cancer patients ((**A**), *n* = 499) who were treated with ((**B**), *n* = 286) or without ET (Supplemental Figure S5C, *n* = 211); specifically, ET treatments included tamoxifen, anastrazole, letrozole, exemestane, and/or fulvestrant. (**C**) The patient data were stratified to evaluate disease-free survival based on high versus low Exosc9 transcript levels; high and low quartile Exosc9 mRNA expression z-scores (relative to normal samples) were used for stratification. Exosc9 mRNA expression z-scores from the TCGA PanCancer Atlas samples were compared between luminal A breast cancer patients who received endocrine therapy and luminal A breast cancer patients who did not receive endocrine therapy. (**D**) Chromatin-bound protein immunoprecipitation for HP1α in TamS-7 (MCF7) and TamR-7 cells. Lysate and immunoprecipitation fractions were then immunoblotted for HP1α, SUMO2/3, and Exosc9. The red arrows represent SUMO-conjugates of HP1α. (**E**) SUMOylated-HP1α to total HP1α protein expression was compared, as well as (**F**) Exosc9 to total HP1α protein expression. (**G**) Tumor spheroids were formed from siRNA-treated TamR-7 cells. The white scale bar indicates 200μm in top two images and 50μm in bottom two images. Spheroid number (**H**) and spheroid diameter (**I**) were compared between siNT and siExosc9 transfected tumor spheroids. (Student’s *t*-test: * *p* value < 0.05; ** *p* value < 0.01).

Breast invasive carcinoma (TCGA, PanCancer Atlas) was utilized to compare BCa patient data. Compared to other BCa types, Exosc9 expression is significantly higher in ER+/HER2- BCa patients (Supplemental Figure S5A). Interestingly, compared to noncancerous mammary epithelial cells, Exosc9 protein expression is reduced in MCF7 cancer cells while Exosc9 protein expression is induced in tamoxifen-resistant (TamR-7) cells (Supplemental Figure S5B). Therefore, we compared disease-free survival of all luminal A BCa patients based on high and low Exosc9 mRNA transcript expression (Figure 4A). No significant difference in disease-free survival was observed between patients with high and low Exosc9 expression in all luminal A BCa patients or patients with non-ET treatment (Supplemental Figures S5C and S6). Disease-free survival is considered when determining treatment effectiveness. When the data was narrowed down to only endocrine therapy treated luminal A BCa patients, disease-free survival was statistically lower in patients with high Exosc9 mRNA transcript expression compared to patients with low Exosc9 mRNA transcript expression (Figure 4B). Additionally, Exosc9 mRNA transcript expression is significantly higher in endocrine therapy treated patients compared to those who did not undergo endocrine therapy (Figure 4C). Furthermore, the ratio of SUMO-HP1a protein expression to unmodified HP1a protein expression in TamR-7 cells is significantly greater than in MCF7 cells (Figure 4D,E). Additionally, Exosc9 protein expression compared to unmodified HP1a protein expression in TamR-7 cells is significantly higher than in MCF7 cells (Figure 4D,F).

We continued in vitro studies by generating tumor spheroids from siRNA (siNT or siExosc9)-treated TamR-7 cells (Figure 4G). The single tumorsphere number was reduced following the knockdown of Exosc9 (Figure 4H). Additionally, a reduction in tumorsphere size was observed following the knockdown of Exosc9 (Figure 4I).

### 3.5. Exosc9 Regulates R-Loops and Telomeric Integrity in ET-Resistant BCa Cells

**Figure 5:**
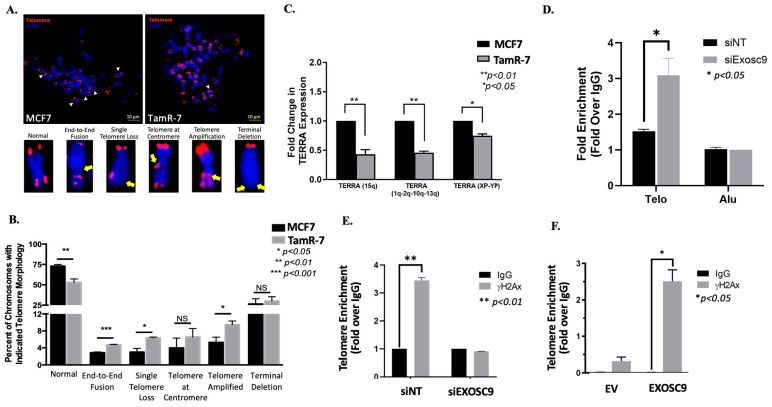
Telo-FISH-stained metaphase chromosomes were evaluated from TamS-7 (MCF7) and TamR-7 cells. (**A**) Representative images are included, which are characteristic of telomere morphology: normal, end-to-end fusion, single telomere loss, telomere at centromere, telomere amplification, and terminal deletion. White arrowheads highlight normal telomeres and yellow arrows indicate telomeric aberration.(**B**) Quantification of telomere morphology compared between TamS-7 and TamR-7. TERRA expression decreased in TamR-7 cells. (**C**) RT-PCR was performed on TERRA (1q-2q-10q-13q), TERRA (15q), and TERRA (Xp-Yp), and normalized to β-actin in TamS-7 and TamR-7 cells. Fold change of TERRA expression was compared between TamS-7 and TamR-7 cells. Data represent the mean ± SEM of three independent experiments (Student’s *t*-test: ** *p-*value < 0.01). (**D**) DNA-RNA enrichment at the telomeres in Exosc9-deficient TamR-7 cells. TamR-7 cells were transfected with either siNT or siExosc9. Chromatin immunoprecipitation was performed using the S9.6 antibody, and enrichment at the telomeres was compared. Data represent the mean ± SEM of three independent experiments (Student’s *t*-test: * *p-*value < 0.05). (**E**) TamR-7 cells were transfected with siRNA-siNT or siExosc9. Cells were then fixed and chromatin immunoprecipitated for γH2Ax. Telomere enrichment was compared to the IgG control (Student’s *t*-test, ** *p-*value < 0.01). (**F**) Noncancerous mammary epithelial cells were treated with either empty vector or overexpression for Exosc9. Cells were then fixed and chromatin immunoprecipitated for γH2Ax. Telomere enrichment was compared to the IgG control. (Student’s *t*-test, * *p-*value < 0.05).

We first checked for telomeric abnormalities in the ET-R BCa cells by fluorescently labeling telomeric DNA, utilizing telomere fluorescence in situ hybridization (Figure 5A). We compared the presence of abnormal morphologies between tamoxifen-resistant (TamR-7) or tamoxifen-sensitive (MCF7) cells. The percentage of chromosomes with overall normal telomere morphology statistically decreased in TamR-7 compared to MCF7. Furthermore, the presence of telomere dysfunction-induced foci was greater in the TamR-7 cells when compared to the MCF7 cells (Supplemental Figure S7). Additionally, the presence of end-to-end fusion, single telomere loss, and telomeres at the centromere abnormal telomere morphology were significantly higher in TamR-7 compared to MCF7.

As previously described, Exosc9-HP1α interaction dictates TERRA levels. Therefore, we compared the mRNA expression of multiple TERRA transcripts (TERRA (15q), TERRA (1q-2q-10q-13q), and TERRA (XP-YP) in TamS-7 (MCF7) and TamR-7 cells (Figure 5C). Interestingly, the TERRA expression of each of the evaluated transcripts markedly decreased in the TamR-7 cells when compared to the TamS-7 cells. This indicates that the presence of Exosc9 reduces the expression of TERRA. We know that TERRA is involved in the formation of telomeric R-loops. The normal expression of Exosc9 in TamR-7 cells is higher compared to TamS-7 cells. We hypothesized that by reducing the expression of Exosc9, TERRA expression would increase in TamR-7 cells, thus increasing telomeric R-loop formation. Indeed, DNA-RNA hybrid formation at the telomeres is significantly higher in the TamR-7 siExoc9 transfected cells compared to the control (Figure 5D).

Since TERRA has a critical role in maintaining telomeric integrity, we assessed the impact of overexpressing Exosc9 in the noncancerous mammary epithelial cells on telomeres. Interestingly, the exogenous expression of Exosc9 resulted in the induction of DNA damage indicated by a greater γH2Ax colocalized signal (Supplemental Figure S8). Additionally, we knocked down Exosc9 in the TamR-7 cells and performed chromatin immunoprecipitation for the presence of phospho-γH2Ax. The telomere enrichment of γH2Ax was significantly higher in the nontargeting treatment when compared to the control, whereas there was virtually no difference in γH2Ax telomere enrichment following the knockdown of Exosc9 (Figure 5E). Interestingly, the noncancerous mammary epithelial cells overexpressed Exosc9, resulting in a significant increase in telomere enrichment γH2Ax expression (Figure 5F). Collectively, these data suggest that the increased expression of Exosc9 at the telomeres results in greater DNA damage.

### 3.6. Exosc9 Levels Dictate Sensitivity to PARP Inhibitors

**Figure 6:**
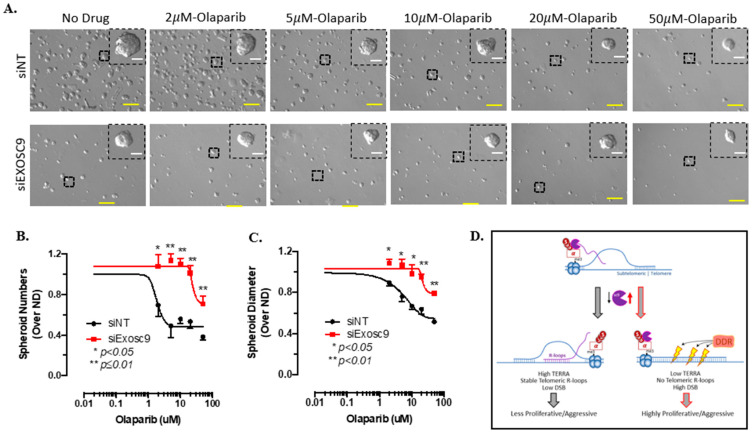
(**A**) Spheroids derived from TamR-7 cells were transfected with siNT or siExosc9 followed by drug treatment with increasing concentrations of the PARP inhibitor, olaparib (no drug (ND), 2 µM, 5 µM, 10 µM, 20 µM, and 50 µM). The yellow scale bar is representative of 200 µm. The white scale bar is representative of 50 µm. Spheroid number (**B**) compared between siNT (black) and siExosc9 (red). Spheroid diameter (**C**) compared between siNT (black) and siExosc9 (red). (**D**) Working Model. See text for description.

PARP, as a major DDR regulator, is an attractive therapeutic target for the treatment of several cancers and is currently being investigated in clinical trials as a monotherapy for breast cancer [28,29,30]. In tamoxifen-sensitive MCF7 cells, the overexpression of Exosc9 supports proliferation. Additionally, Exosc9-induced MCF7 cells acquire resistance to tamoxifen and become increasingly sensitive to olaparib treatment (Supplemental Figure S9). Therefore, we evaluated the effect of PARPi olaparib on tumor spheroid formation in tamoxifen-resistant cells with a targeted reduction in Exosc9 (Figure 6A). With increasing concentrations of olaparib, tumor formation significantly decreases in the spheroids derived from tamoxifen-resistant cells. Comparatively, nondrug treated tumor spheroid formation was lower in siExosc9 transfected cells. However, increasing the concentrations of olaparib had less of an effect of decreasing tumor spheroid formation in those cells targeting a reduction in Exosc9, with median IC50 values for olaparib at 1.808 μM and 23.66 μM in siNT versus siExosc9-treated TamR-7 cells, respectively (Figure 6B). Furthermore, increasing the drug concentration decreased the individual tumorsphere size in the nontargeting cells, but this decreased size was not observed in the siExosc9 cells, with median IC50 values for olaparib at 2.871 μM and 21.46 μM in siNT versus siExosc9-treated TamR-7 cells, respectively (Figure 6C). Overall, olaparib had less of an effect on tumorspheres, with reduced Exosc9 expression.

## 4. Discussion

### 4.1. TERRA Degradation

Few studies define the mechanisms for cell cycle-dependent TERRA degradation, especially in human cells. One report highlights a role for mRNA surveillance nonsense RNA-mediated decay (NMD) factors. Specifically in shRNA-treated telomerase-positive HeLa cells, NMD factors displace TERRA from telomeres without directly altering total TERRA lncRNA levels [31]. Similarly, RNA endonuclease H1 (RNase H1) removes transgenic TERRA from telomeric R-loops in transfected HeLa cells [1]. However, RNaseH1’s resolution of R-loops is stress-induced and independent of the cell cycle [32]. In yeast cells, 5′ to 3′ RNA exonuclease, ribonucleic acid trafficking 1p (Rat1p), and RNaseH2 degrade TERRA in a cell cycle-dependent manner [33,34]; however, an analogous cell cycle mediated TERRA degradation was not proposed for human cells.

In human cells, we now present the RNA exosome component Exosc9 as a novel regulator of TERRA and telomeric R-loops. Specifically, Exosc9 is enriched at the telomeres in a cell cycle-dependent manner, degrades TERRA, and consistently dictates telomeric R-loops in human mammary cells (Figure 2, Figure 3 and Figure 5). Other components of the RNA exosome are known to direct R-loops outside the telomeres in additional cells. The depletion of Exosc3 or Exosc10 leads to the accumulation of antisense-RNA R-loops in differentiated B cells and pluripotent embryonic stem (ES) [10]. Exosc10 is the established catalytic subunit, whereas Exosc9 is one of six core components of the RNA exosome [22]. Exosc9 is enriched at telomeres in mammary epithelial cells. This is consistent with reported studies in HEK293 and HELA cells [35]. Unlike Exosc9, neither Exosc10 nor another core component, Exosc4, is enriched at the telomere (Figure 1C). The core exosome subunits in eukaryotes exhibit independent catalytic activity [27]. Consistently, recombinant Exosc9 selectively degrades RNA, including chromosome-specific TERRA (Figure 3C–F).

SUMO PTM supports protein–protein interactions through both covalent and noncovalent SUMO binding, particularly as part of the DNA damage response network [36]. Interestingly, the yeast ortholog of Exosc9, Rrp45, interacts with an RNA:DNA helicase SETX at DNA damage foci [37]. Compellingly, this Rrp45-SETX interaction requires the prior SUMO-modification of SETX’s and Rrp45’s C-terminal SUMO-interaction motif (SIM). Similarly, the SUMOylation of HP1α is required for the interaction with Exosc9 (Figure 1D–F). We expect that because HP1α SUMOylation is dynamic and cell cycle-dependent, this SUMO-PTM supports the transient enrichment of Exosc9 at telomeres in normal cells. Consistently, HP1α SUMOylation, the telomeric enrichment of Exosc9, and a reduction in TERRA occur during the S-G2 phase transition. Hence, for the first time, this study highlights the SUMO regulatory control of lncRNA TERRA clearance.

### 4.2. Exosc9 Levels and BCa Therapy

Exosc9 is upregulated in HR+ BCa treated with ET-R, and the elevated Exosc9 levels in these patients correlate with a lower probability of disease-free survival (Figure 4A–C). Others report that high Exosc9 activity correlates with poor prognosis in adrenocortical, lung, and pancreatic adenocarcinoma [30]. In addition, Exosc9 is one of the genes identified to support lung and breast cancer cell growth during hypoxia [38]. The depletion of Exosc9 induces excess APOBEC3G mRNA expression and, subsequently, reduces P-body formation and stress resistance in breast cancer cells [39].

Exosc9 expression impacts the proliferation and aggressiveness of ER+ BCa cells (Figure 6D). Low Exosc9 levels support TERRA accumulation and consistent telomeric R-loop formation. TERRA slows telomere replication by (1) sequestering and inhibiting telomerase and/or (2) supporting stable R-loops to further impede telomerase enrichment at the telomeres [6]. Hence, BCa cells with lower Exosc9 levels are less proliferative, and the tumors are less aggressive.

Inversely, elevated Exosc9 actively degrades TERRA, prevents TERRA enrichment at the telomeres, inhibits telomeric R-loops, and thereby potentiates telomerase-dependent telomere replication. Concurrently, the loss of TERRA and its protective structure enhances DNA damage at the chromosome ends. Others have also demonstrated the correlation between TERRA expression and DNA damage at the telomeres. The depletion of TERRA by siRNA or antisense oligonucleotide containing locked nucleic acid (ASO-LNA) initiates damage at the telomeres and prompts telomere dysfunction-induced foci (TIF) formation [3,7]. The deletion of the TERRA locus from telomere 20q using clustered, regularly interspaced short palindromic repeats and CRISPR-associated protein 9 (CRISPR/Cas9) increases DNA damage at multiple chromosome ends [40]. However, DDR machinery is frequently upregulated in ET-R BCa, and DNA strand breaks (DSBs) can be efficiently repaired. Consistently, the Exosc9-high BCa cells exhibit a dependency on the DDR system to proliferate. Consequently, BCa cells with elevated Exosc9 are more sensitive to DDR inhibitors like the PARP inhibitor olaparib.

Olaparib is an FDA-approved monotherapy for HR+ BCa with a germline BRCA mutation; patients with BRCA mutations exhibit impaired DDR, high genotoxic stress, and aggressive BCa [41]. Additional clinical trials to test the efficacy of olaparib as a monotherapy for non-BRCA DDR gene mutations in HR+ BCa are ongoing. As DNA damage accumulates with mainstay tamoxifen ET in HR+ BCa patients, it is not surprising that olaparib is being proposed as a next-line monotherapy for patients insensitive to ET [42]. In this regard, Exosc9 may serve as a promising biomarker for predicting responsiveness to olaparib and other PARP inhibitors for ET-R HR+ BCa. Specifically, patients with elevated Exosc9 expression would be good responders to PARP inhibitors.

## 5. Conclusions

This study establishes a novel mechanism for regulating TERRA degradation on telomeres in mammary epithelial cells. Specifically, during the S/G2 phase, Exosc9 is recruited to the telomeres via interaction with SUMOylated HP1α to initiate TERRA degradation at the telomeres. Exosc9 levels are elevated and drive metastatic disease in those patients with HR-positive BCa treated with conventional endocrine therapy. With the onset of ET-R, elevated Exosc9 disrupts telomeric R-loops to potentiate DNA damage at the telomeres. Consequently, Exosc9 predicts the response to DDR-targeted therapy, i.e., PARP inhibitors.

## Data Availability

The data presented in this study are available within the article or in the Supplemental Material.

## Supplementary Materials

The following supporting information can be downloaded at https://www.mdpi.com/article/10.3390/cells12202495/s1, Figure S1: Alu Control and Transfection Efficiency; Figure S2: Synchronization of Cells and TERRA Expression During Cell Cycle in Noncancerous Mammary Epithelial Cells; Figure S3: Transfection Efficiency and Total RNA Levels in the Presence/Absence of Exosc9; Figure S4: TERRA Expression is Normalized to GAPDH and 18S in the Knockdown and Overexpression of Exosc9 and SUMO-HP1α Reduced TERRA Expression; Figure S5: Induction of EXOSC9 and SUMO-HP1a in ET-resistant BCa; Figure S6: EXOSC9 levels and BCa Patient Overall Survival with Select Anti-Cancer Therapy; Figure S7: TIFs Foci at Telomerers in ETS vs ETR; Figure S8: TIFs Foci at Telomeres in the Overexpression of Exosc9 in noncancerous Mammary Epithelial Cells; Figure S9: Cell Viability in Olaparib-Treated Tamoxifen Sensitive cells and Table S1: Primer Sequences. References [43,44] are cited in the supplementary materials.
