# Supplementary material for: Exosc9 Initiates SUMO-Dependent lncRNA TERRA Degradation to Impact Telomeric Integrity in Endocrine Therapy Insensitive Hormone Receptor-Positive Breast Cancer
Source: Cells. 2023 Oct 20;12(20):2495. doi: 10.3390/cells12202495 (PMC10605189; doi:10.3390/cells12202495)
Supplement: Supplementary file 1 [file cells-12-02495-s001.zip › cells-2587283-supplementary.pdf]

Supplemental Figure S1: Alu Control and Transfection Efficiency.

A.

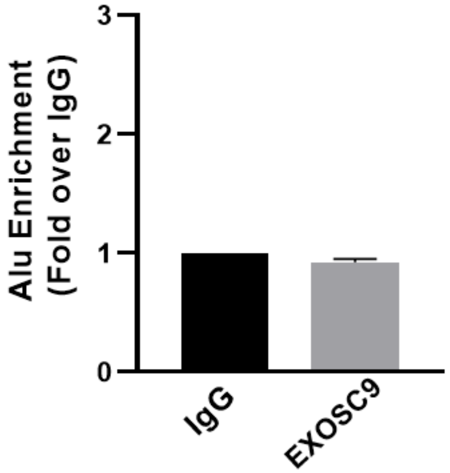

B.

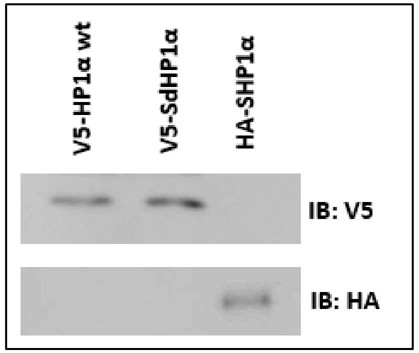

Supplemental Figure S2: Synchronization of Cells and TERRA Expression During Cell Cycle in Noncancerous Mammary Epithelial Cells.

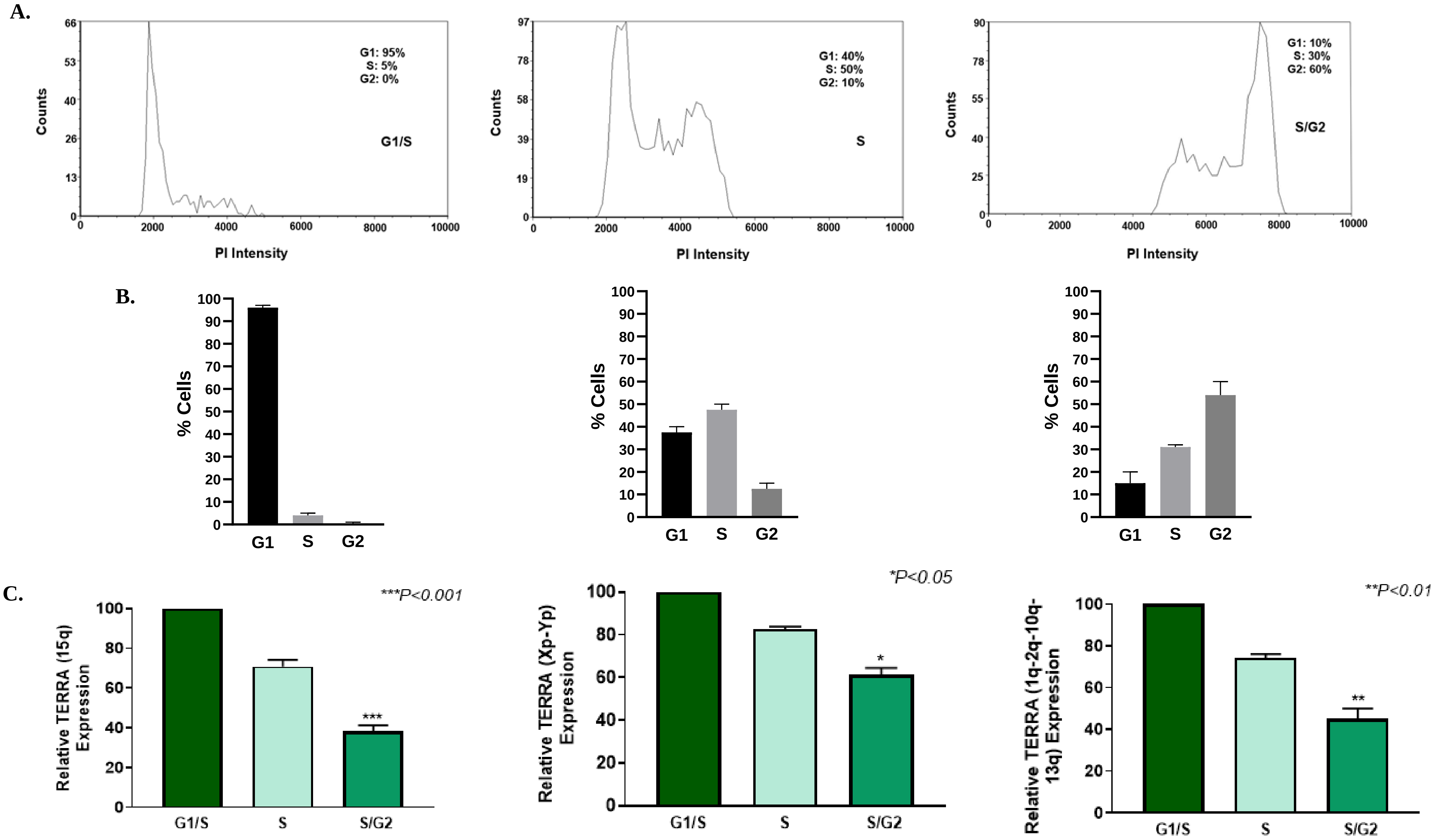

Supplemental Figure S3: Transfection Efficiency and Total RNA Levels in the Presence/ Absence of Exosc9.

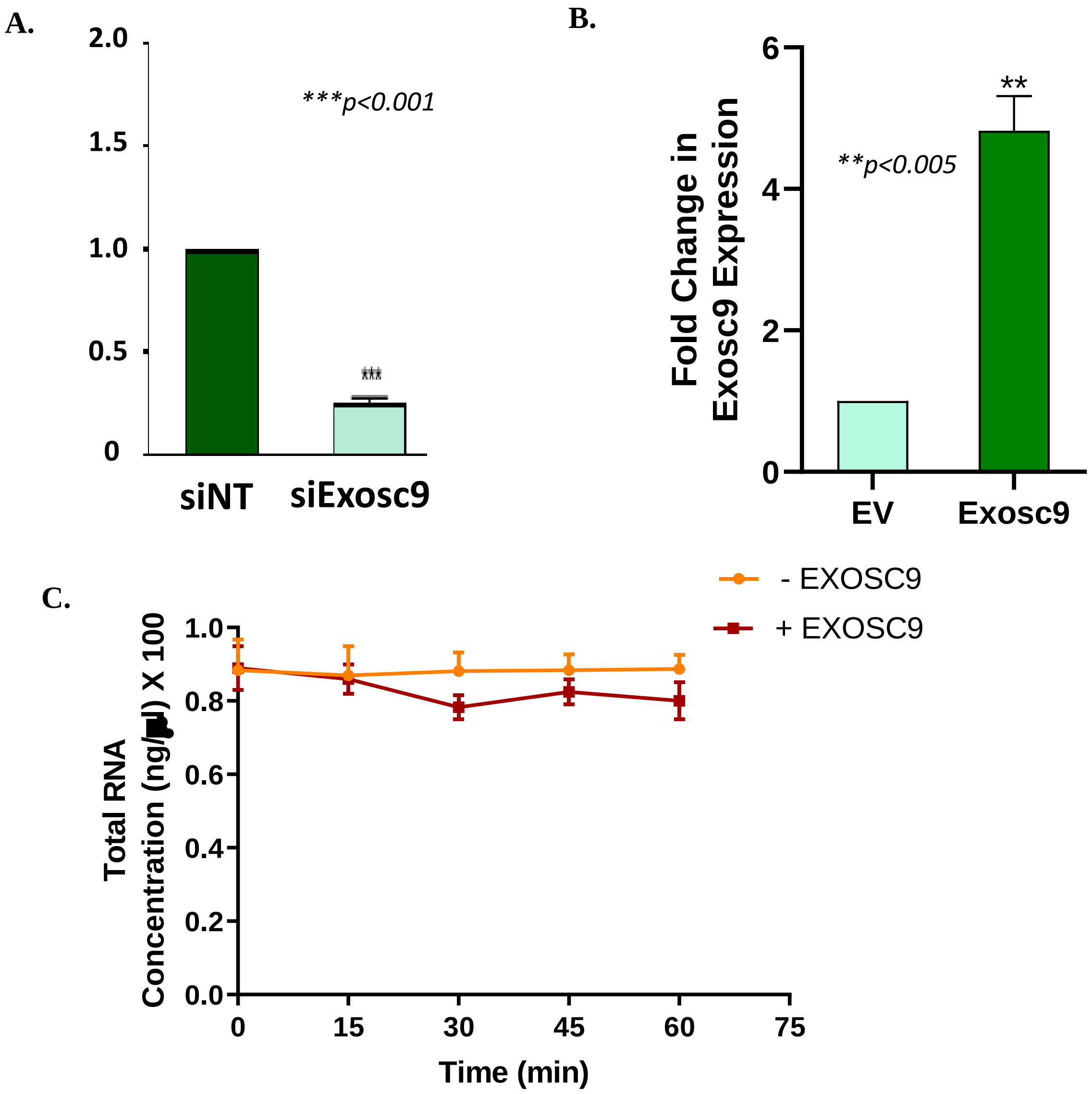

Supplemental Figure S4: TERRA Expression is Normalized to GAPDH and 18S in the Knockdown and Overexpression of Exosc9 and SUMO-HP1α Reduced TERRA Expression.

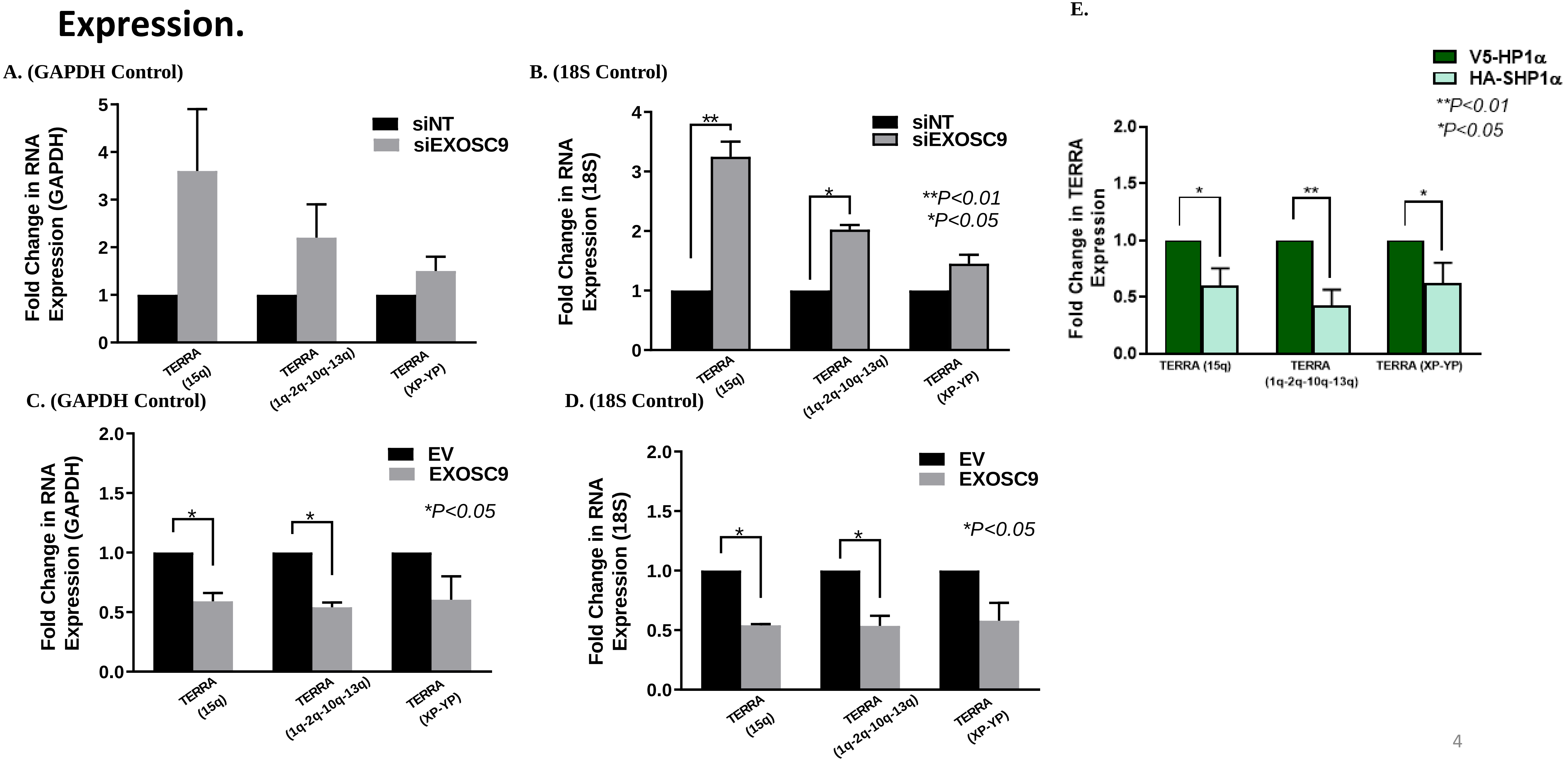

Supplemental Figure S5: Induction of EXOSC9 and SUMO-HP1α in ET-resistant BCa.

A.

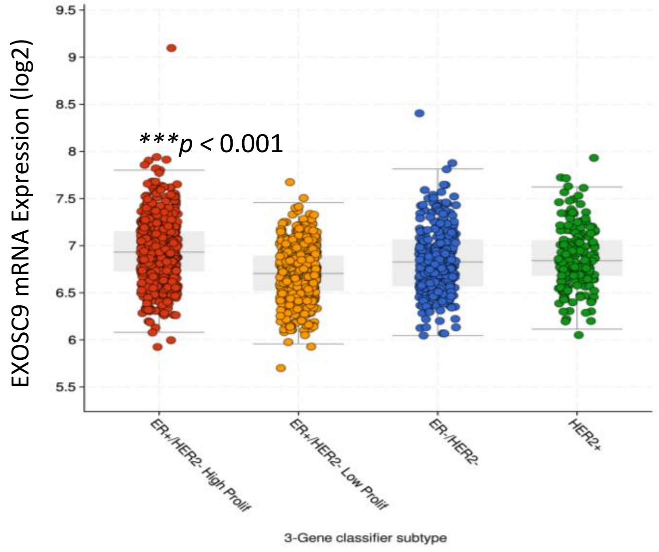

B.

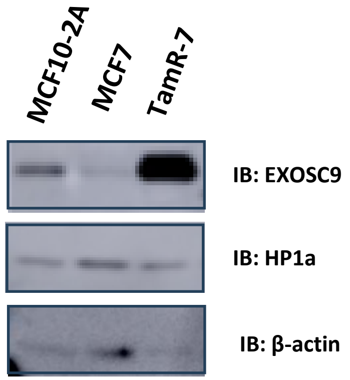

C.

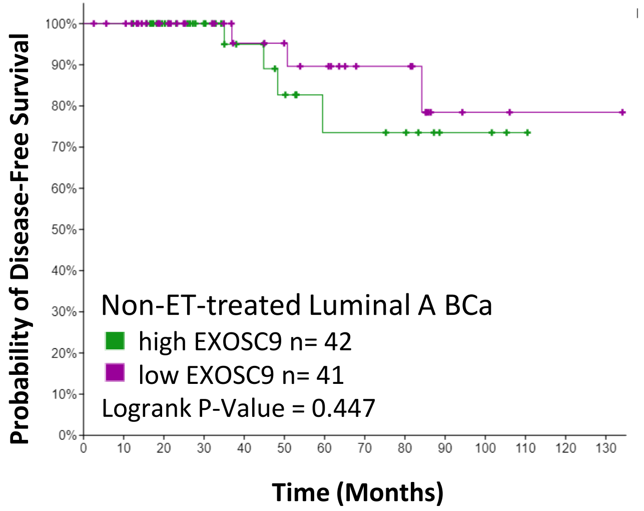

Supplemental Figure S6: EXOSC9 levels and BCa Patient Overall Survival with Select Anti-Cancer Therapy.

A. All BCa Patients TCGA

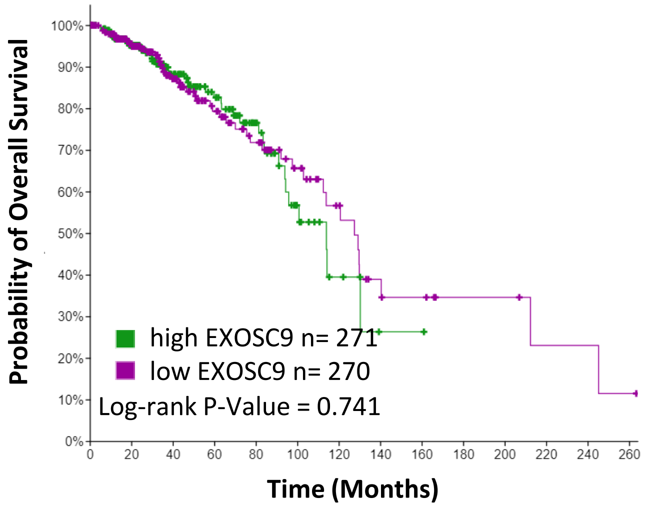

B.

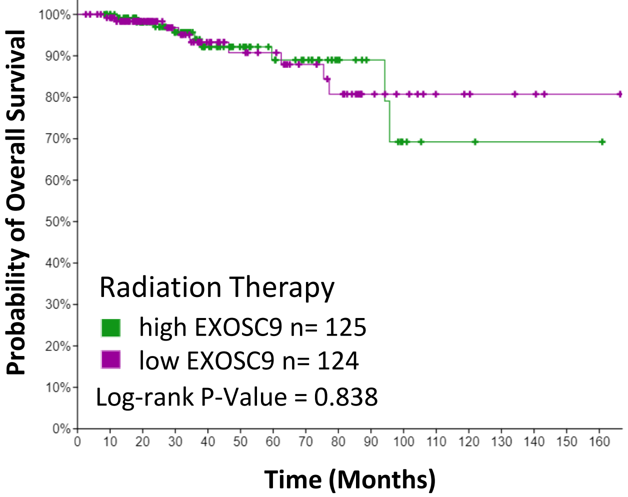

C.

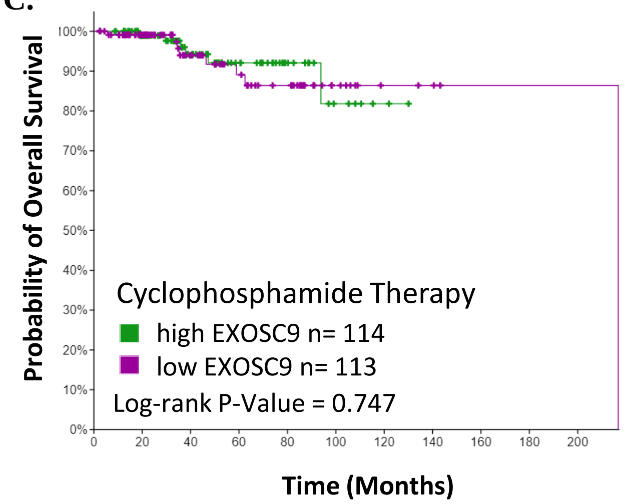

D.

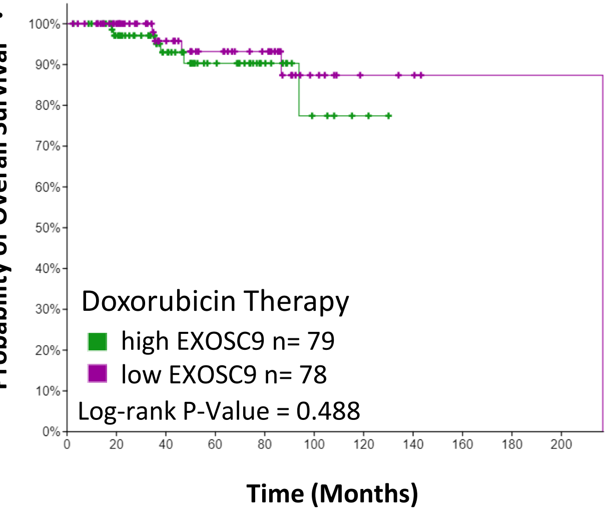

Supplemental Figure S7: TIFs Foci at Telomerers in ETS vs ETR.

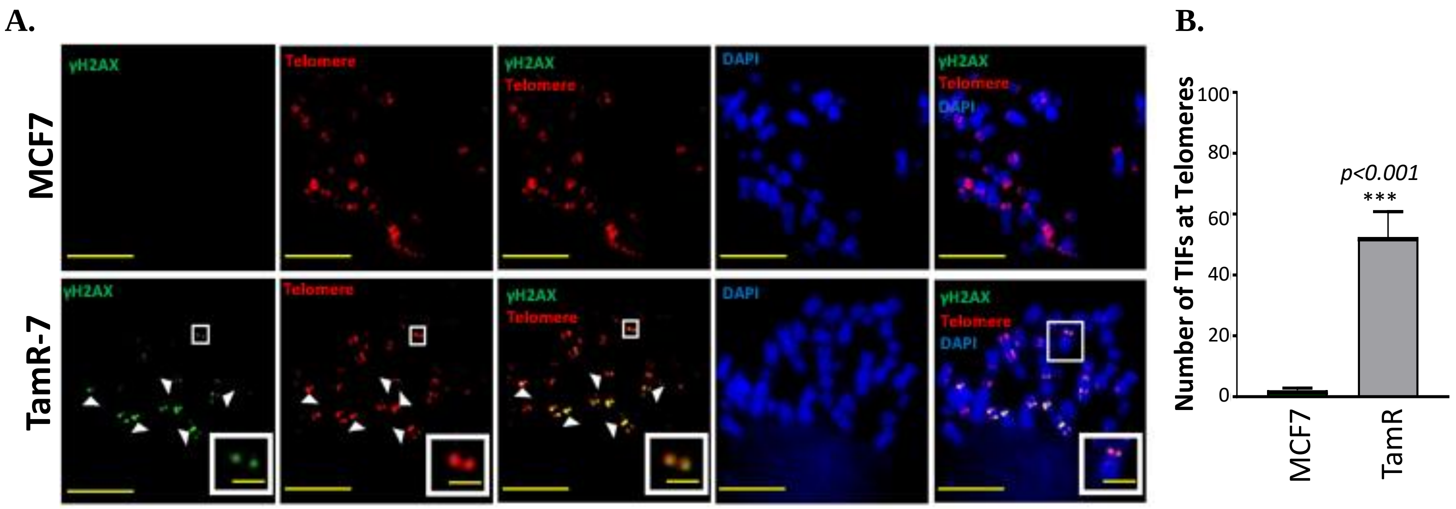

Supplemental Figure S8: TIFs Foci at Telomeres in the Overexpression of Exosc9 in noncancerous Mammary Epithelial Cells.

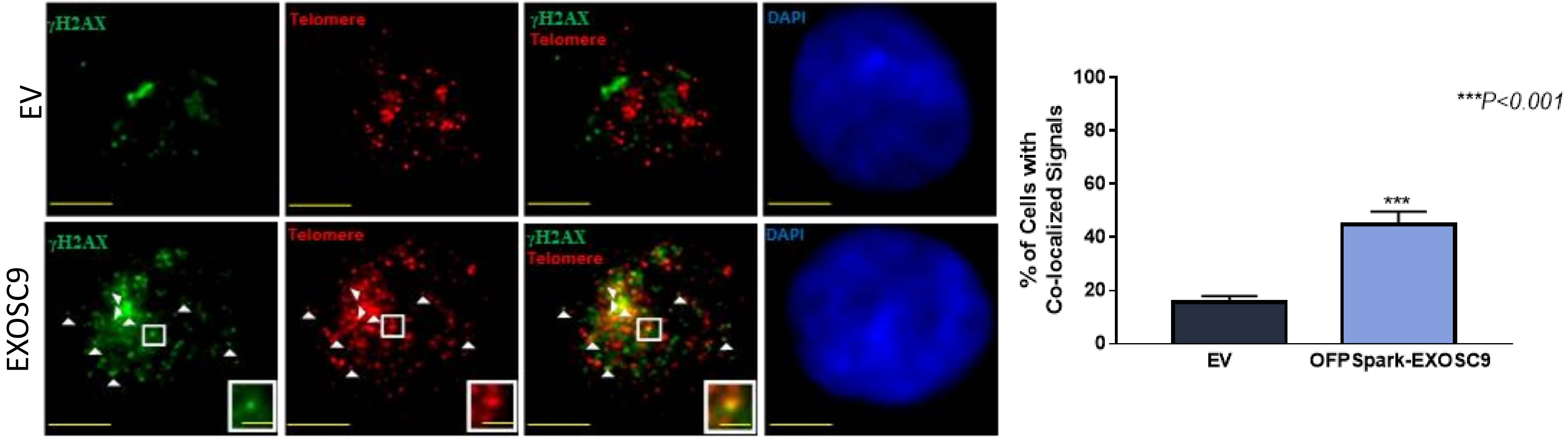

Supplemental Figure S9: Cell Viability in Olaparib-Treated Tamoxifen Sensitive cells.

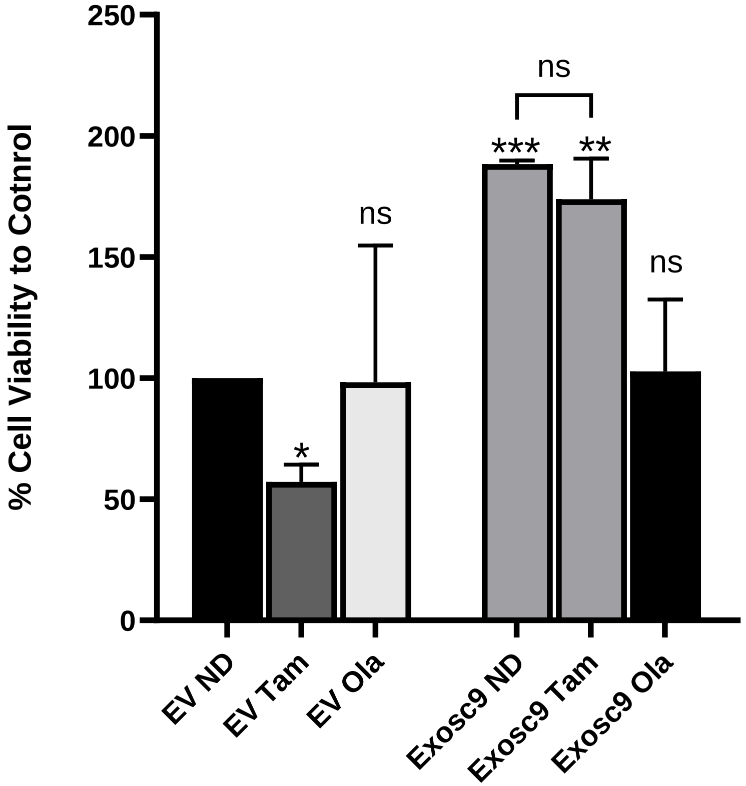

Supplemental Table S1: Primer Sequences.

Target	Primer Sequence	Reference
**TERRA (1q-2q-10q-13q)**	**Forward:** 5’-'GAATCCTGCGCACCGAGAT-3’<br>**Reverse:** 5’-CTGCACTTGAACCCTGCAATAC-3’	[43]
**TERRA (15q)**	**Forward:** 5’-CAGCGAGATTCTCCCAAGCTAAG-3’<br>**Reverse:** 5’-AACCCTAACCACATGAGCAACG-3’	[2]
**TERRA (XpYp)**	**Forward**: 5’-GCAAAGAGTGAAAGAACGAAGCTT-3’<br>**Reverse:** 5’-CCCTCTGAAAGTGGACCAATCA-3’	[2]
**XIST**	**Forward:** 5’- CTTGAAGACCTGGGGAAATCCC-3’<br>**Reverse:** 5’- TGTCAATCTAAAGGTAACCGGC-3’	[8]
**HOTAIR**	**Forward:** 5’- CAGTGGAATGGAACGGATTT-3’<br>**Reverse:** 5’- TCAGACTCTTTGGGGCCTTAC-3’	[8]
**FIBRONECTIN**	**Forward:** 5’-CCGCCGAATGTAGGACAAGA-3’<br>**Reverse:** 5’-TGCCAACAGGATGACATGAAA-3’	[20]
**b-actin**	**Forward:** 5’-CCATCAACGACCCCTTCATTGACC-3’<br>**Reverse:** 5’- TGGTTCACACCCATCACAAACATG-3’	[20]
**Exosc9**	**Forward:** 5’-TCG GGC AAC AGA AGG TAT TC-3’<br>**Reverse:** 5’-GTC CAT AGC ACA GGT GTA GAA A-3’	
**Telomere primer for TELO-ChIP**	**Forward:** 5’-ACACTAAGGTTTGGGTTTGGGTTTGGGTTTGGGTTAGTGT-3’<br>**Reverse**: 5’-TGTTAGGTATCCCTATCCCTATCCCTATCCCTATCCCTA-3’	[44]

43. Diman, A., et al., *Nuclear respiratory factor 1 and endurance exercise promote human telomere transcription.* Science Advances, 2016. **2**(7): p. e1600031.

44. Cawthon, R. *Telomere measurement by quantitative PCR.* Nucleic Acids Research, 2002. **30**(10): p.e47.
